# Professional biofilm management during supportive periodontal therapy—a longitudinal observational study

**DOI:** 10.1186/s12903-025-06642-7

**Published:** 2025-07-30

**Authors:** Miriam Cyris, Georgina Hach, Christof E. Dörfer, Karim Fawzy El-Sayed, Christian Graetz

**Affiliations:** 1 Clinic of Conservative Dentistry and Periodontology, University of Kiel, Kiel, Germany; 2 Oral Medicine and Periodontology Department, Faculty of Dentistry, Cairo University, Giza, Egypt

**Keywords:** Air-flowing, Oral biofilm, Supportive periodontal care, Prophylaxis, Periodontitis, Rubber cup

## Abstract

**Background:**

Professional mechanical biofilm reduction represents the cornerstone measure during supportive periodontal therapy (SPT). Conventionally, rotating polishing rubber cups (RCs) and brushes with polishing paste or air-polishing (AP) devices using low-abrasive powders can be used. This study aimed to evaluate the effectiveness of both methods in periodontitis patients in a university SPT setting.

**Methods:**

Patients diagnosed with periodontitis who attended regular SPT at the Department of Conservative Dentistry and Periodontology at the University Hospital Schleswig-Holstein, Kiel campus, at least once a year between 2018 and 2023 were included. Clinical parameters such as number of teeth and pocket probing depth (PPD) were recorded at T1 (first documented SPT session) and T2 (last documented SPT session).

**Results:**

A total of 430 patients (AP/RC: *n* = 152/*n* = 278) with an average age of 60.7(11.5) years, were included. Most patients had Stage III (AP/RC: 56.6%/56.8%) and Grade B (AP/RC: 52%/64.4%) periodontitis. The treatment time was 77.9(21.0) min and did not differ between groups (*p* = 0.378). No significant differences were found in the number of sites with PPD ≤ 4 mm at T1 or T2 (*p* > 0.05). Sites with PPD ≥ 5 mm differed significantly at T1 (AP: 8 [4–16], RC: 6 [3–12]; *p* = 0.002) but not at T2 (AP: 6 [3–13], RC: 5.5 [3–11]; *p* = 0.104). No significant intergroup differences were notable regarding stability, improvement, or deterioration of sites with PPD ≥ 5 mm over time. However, the AP group had significantly more multirooted teeth with Stage III furcation involvement regardless of bleeding on probing (BOP) at T1 (AP: 2 [1–5], RC: 1 [1–3]; *p* = 0.046) but not at T2. AP demonstrated a significant advantage in preventing deterioration of PPD ≥ 5 mm in molars (AP: 48 [45.3%], RC: 62 [33.3%]; *p* = 0.027).

**Conclusions:**

Both methods of professional biofilm removal are similarly effective in terms of stabilizing or improving periodontal sites with PPD ≥ 5 mm when performed regularly. However, in molars with furcation involvement, RC intervention showed more favorable outcomes compared to AP, particularly in preventing the deterioration of sites with PPD ≥ 5 mm, in this study cohort treated in a specialized university-based SPT setting.

**Trial registration:**

The study was retrospectively registered in the DRKS—German Clinical Trials Register (https://www.drks.de) with the registration-ID DRKS00037021 (22/05/2025).

## Background

Periodontitis, one of the world’s most common chronic diseases [1], affects more than 50% of the global population, becoming more prevalent with increasing age [2]. It represents a chronic multifactorial inflammation of the periodontium associated with microbial dysbiosis; if left untreated, it leads to significant periodontal attachment damage and even tooth loss [3].

As such, destruction is irreversible [4], and the systematic inflammatory response and extent of the disease are underestimated. Consequently, the provision of effective and immediate professional active periodontal treatment (APT) and a subsequent integration of the patients into lifelong supportive periodontal therapy (SPT) are mandatory. Such SPT includes oral hygiene instructions as well as professional mechanical plaque removal (PMPR) and subgingival instrumentation of sites with PPD ≥ 5 mm–4 mm with bleeding on probing (BOP) to maintain periodontal stability, in line with a documentation of pocket probing depths (PPDs) and BOP. A wide range of instruments have been used for supra- and subgingival PMPR, including sonic and ultrasonic scalers as well as hand instruments. Additionally, rotating rubber cups (RCs) or brushes with polishing paste or air-polishing (AP) devices, using low-abrasive powders, are used to remove non-mineralized biofilm, though conventional polishing with polishing paste is limited to supragingival areas, whereas powder-water jet devices with low-abrasive powders can operate supra- and subgingivally [5]. Such subgingival application has been demonstrated to have a positive effect on the subgingival composition of the microbiome [6], with glycine powder having the ability to reduce the number of periodontal pathogenic bacteria, including *Porphyromonas gingivals* [7].

In recent years, various randomized controlled trials and systematic reviews have investigated the clinical efficacy of air-polishing (AP) and conventional polishing techniques (e.g., RC) during SPT. A comprehensive systematic review concluded that AP is an effective alternative to conventional methods, demonstrating comparable clinical outcomes while improving patient comfort and reducing treatment-related discomfort [8]. These findings were supported by Kaur et al., who observed similar levels of plaque and stain removal for both techniques, though with a slight preference for RC in terms of soft tissue tolerability [9]. More recently, Stähli et al. evaluated a novel SPT protocol in a randomized clinical trial and highlighted the importance of individualized instrument selection based on site-specific anatomical challenges [10]. Specifically, studies focusing on molar furcation sites have suggested that AP performance may be limited by complex root anatomy. Ulvik et al. demonstrated that while erythritol-based AP is effective in shallow furcation-involvement, conventional debridement with curettes or ultrasonic devices may offer superior clinical attachment gain in deeper lesions [11]. A long-term retrospective study further confirmed the clinical safety and effectiveness of repeated subgingival AP application in SPT but also emphasized the need for tailored indications, particularly in furcation-involved teeth [12].

Therefore, the study aimed to evaluate the effectiveness of both methods in periodontitis patients treated within a university-based SPT setting. We hypothesized, that patients treated according to the test procedure would exhibit a significantly lower number of sites with PPD ≥ 5 mm after the observation period. This research was conducted from January 2018 until December 2023, with patients participating in the treatments at least once a year.

## Methods

### Study design

The study included a cohort of 430 patients diagnosed with periodontitis who received a systematic APT and participated regularly in SPT at least once a year between January 2018 and December 2023, in the Clinic for Conservative Dentistry and Periodontology at University Hospital Schleswig-Holstein, Kiel campus, Germany. During this period of SPT, clinical parameters—including number of teeth, pre-existing systemic conditions (diabetes, cardiovascular diseases or others), pocket probing depth (PPD), furcation involvement (FI), BOP, degree of mobility, smoking status (yes/no), plaque index (%) [13], and dental status at the time of T1 (first documented SPT session) and T2 (last documented SPT session)—were documented for each patient. The dental status documented the presence of caries (yes/no), the quality of restoration (sufficient/insufficient), and the type of restoration (conservatively or prosthetically). Implants were also recorded as part of the findings but were not included in the analyses. In addition, each patient was reclassified according to the current classification [14] based on clinical parameters and the most currently available panoramic radiograph (not older than 5 years) at T1. Additionally, the average duration of treatment was documented; therefore, the maximum length of 30 sessions could be used to calculate the average. All clinical examinations were performed by experienced periodontal specialists and followed the treatment guidelines of the department as previously described in detail [15, 16]. These include an internal calibration for periodontal measurements, which is conducted regularly as part of quality assurance [17]. In addition, all clinicians take part in regular trainings as part of the institution’s quality assurance program. A measurement deviation of ± 1 mm for the probing depths (PPD) is considered acceptable.

Inclusion criteria required at least one documented SPT session per year and complete clinical records at both T1 and T2. Patients were excluded from the study if they did not participate in the study at least once a year during the observation period of five years, had incomplete data at either T1 or T2, declined consent for data use, or died during the observation period. No imputation of missing data was performed.

The cohort was divided into two groups; the test group received mainly AP using low-abrasive powder (glycine or erythritol) and the control group received a polishing with an RC and polishing paste with different degrees of abrasion during SPT.

Due to the observational design of the study, treatment allocation was not randomized but determined by routine clinical practice and prevailing clinical preference. Full blinding of clinicians was not feasible. However, a degree of independence was preserved; dental hygienists performed the SPT, while periodontal assessments were conducted separately by the investigators. Furthermore, the statistician responsible for the analysis was blinded to group allocation to reduce the risk of detection and analysis bias.

### Protocols for supportive periodontal therapy

To guide and support biofilm removal, all teeth were covered with a disclosing agent to determine the individual’s plaque control level, followed by the delivery of oral hygiene instructions and the removal of supra- and subgingival mineralized and non-mineralized biofilm. For the subgingival instrumentation of tooth surfaces with PPD of ≥ 4 mm and BOP or PPD of ≥ 5 mm, sonic scalers (Proxeo, W&H, Bürmoos, Austria) with a slime-line tip (1AP, W&H), ultrasonic scalers with a slime-line tip (PERIO SLIM PIEZON PS, EMS, Nyon, Switzerland), or manual instrumentation (Gracey curettes, American Eagle Instruments, Missoula, MT, USA) were used.

### Test group (air polishing)

The final biofilm removal in the AP group was performed using the Prophylaxis Master (E.M.S. Electro Medical Systems S.A., Nyon, Schweiz) with erythritol powder (AIRFLOW PLUS^®^ Pulver E.M.S. Electro Medical Systems S.A., Nyon, Schweiz) or the Prophy-Mate neo (NSK Europe GmbH, Eschborn, Deutschland) also with erythritol powder or, less frequently, with glycine powder (PROPHYflex Perio Powder, KaVo Dental, Biberach, Germany). Both erythritol and glycine powder are low-abrasive, but erythritol has a smaller particle size (14 μm, on average) than glycine powder (25 μm, on average).

### Control group (rubber cup)

The final biofilm removal in the RC group was performed mainly using an RC (Edenta AG, Schweiz) with a low degree of hardness and polishing pastes with different levels of abrasion. A range of colors were used to code the degree of abrasion (relative dentin abrasion [RDA] values ranging from 40 to 250 [ProphyCare Prophy-Paste, CCS, Directa, Kümmersbruck, Germany]) for each patient. The yellow prophy paste had the lowest RDA with 40 and a particle size of 2 μm, followed by the pink (RDA 120), green (RDA 170), and blue prophy pastes (RDA 250). The blue paste also had a particle size of 125 μm. In the functional area of periodontology, the green polishing paste (RDA 170) is predominantly used.

### Outcome variables

#### Statistical analysis

A statistical analysis of the measurements was performed with a statistical software (IBM SPSS Statistics for Windows, Version 23.0. Armonk, NY: IBM Corp). A formal a priori sample size calculation was not conducted, as all eligible patients fulfilling the inclusion criteria were consecutively enrolled from the available database. The final sample size of 430 patients allowed for meaningful subgroup analyses. Differences between the groups were analyzed using the Mann–Whitney U test, and intragroup differences were assessed with the Wilcoxon signed-rank test. For both between-group and within-group comparisons, effect sizes were calculated as Cohen’s *d*. For cross-tabulations, the chi-square test was applied, and an effect size was determined using Cramér’s *V*. Statistical significance was assumed at *p* ≤ 0.05.

## Results

The study included 430 patients (AP/RC: *n* = 152/*n* = 278) with an average age of 60.7 (11.5) years. The majority had Stage III (AP/RC: 56.6%/56.8%) and Grade B periodontitis (AP/RC: 52%/64.4%), with a mean bone% over 56.5% (17.1) in the AP group and 53.7% (16.5) in RC group. During the observation period, we found a very low tooth loss for both groups (AP/RC: *n* = 0 [0–2]/ / *n* = [0–1]/), with the following values for each tooth region: (AP/RC: anterior teeth 0 [0–0]/0 [0–0], premolars 0 [0–0]/0 [0–0], molars 0 [0–1]/0 [0–1]). The treatment duration averaged 77.9 (21.0) minutes and demonstrated no significant difference between groups (*p* = 0.378; see Table 1).

**Table 1:** Patients’ characteristics in air-polishing and rubber cup groups at baseline

Baseline characteristics	Test group (AP)	Control group (RC)	Total
Gender (*N* [%])			
Male	68 (44.7%)	10**3** (37.1%)	171 (39.8%)
Female	84 (55.3%)	175 (62.9%)	259 (60.2%)
Age in years (Mean [*SD*])	59 (10.1)	61.7 (12.1)	60.7 (11.5)
Smoker/ Non-smoker (*N* [%]) T1 T2	32 (21.1%)/ 120 (78.9%) 26 (17.1%)/ 126 (82.9%)	36 (12.9%)/ 242 (87.1%) 33 (11.9%)/ 245 (88.1%)	68 (15.8%)/ 362 (84.2) 59 (13.7%)/ 371 (86.3)
Plaque Index (Mean [*SD*]) T1 T2	27.8 (24.2) 55.3 (6.0)	30.3 (31.2) 66.2 (28.7)	29.6 (29.5) 60.8 (20.8)
Recall period in days (Mean [*SD*])	73 (48%)	73 (48%)	1936.6 (1764.2)
Treatment time in minutes (Mean [*SD*])	79.1 (21.5)	77.2 (20.7)	77.9 (21) *
Number of visits (Median [IQR])	9 (7–10)	9 (7–10)	9 (7–10)
Periodontitis stage (*N* [%])			
Stage II	6 (3.9%)	19 (6.8%)	25 (5.8%)
Stage III	86 (56.6%)	158 (56.8%)	244 (56.7%)
Stage IV	60 (39.5%)	101 (36.3%)	161 (37.4%)
Periodontitis grade (*N* [%])			
Grade A	0 (0%)	1 (0.4%)	1 (0.2%)
Grade B	79 (52%)	179 (64.4%)	258 (60%)
Grade C	73 (48%)	98 (35.3%)	171 (39.8%)
Periodontitis extension (*N* [%])			
Localized	0 (0%)	1 (0.4%)	1 (0.2%)
Generalized	152 (100%)	277 (99.6%)	429 (99.8%)
Number of teeth (Median [IQR])			
T1	24 (21–27)	24 (21–27)	24 (21–27)
T2	22 (19.3–27)	23 (19–26)	23 (19–26)
Bone% at T1 (Mean [*SD*])	56.6 (17.1)	53.7 (16.5)	54.7 (16.7)

* *p*-value: 0.378 (Significant at *p* ≤ *0.05)*

IQR: Interquartile range, SD: Standard deviation

### Pocket probing depth (PPD) changes over time

In both groups, the number of anterior sites with PPD 0–3 mm remained unchanged at T2 (*p* > 0.05), while a significant reduction was observed in premolars and molars (AP: *p* = 0.005 and *p* = 0.001; RC: *p* = 0.028 and *p* < 0.001). For PPD 4 mm, no significant change was found in the AP group (*p* > 0.05), whereas the RC group exhibited a significant reduction in all regions, particularly in molars (*p* = 0.041). Sites with PPD ≥ 5 mm remained stable in both groups (*p* > 0.05). For PPD ≥ 6 mm, no significant changes were observed in the AP group (*p* > 0.05), while molar sites in the RC group indicated a significant reduction (*p* = 0.030; see Table 2).

**Table 2:** Descriptive statistics and results of Wilcoxon signed-rank test for the changes in sites with pocket probing depth at T1 and T2 for different tooth types in air-polishing and rubber cup groups

PPD	Group	Tooth type	T1			T2			*P*-value
			N	Median	IQR	N	Median	IQR	
0–3	AP	Anterior	151	65	56–71	151	66	54–71	0.222
		Premolar	150	34	23–42.3	150	33	23–42	0.005*
		Molar	145	23	14–32.5	145	22	13–29.5	0.001*
	RC	Anterior	278	65	55–71	278	65.5	55.8–71	0.986
		Premolar	277	36	27–43	277	36	25–42	0.028*
		Molar	268	24.5	16–32	265	22	14–31	< 0.001*
	Total	Anterior	429	65	55–71	429	66	55–71	0.389
		Premolar	427	36	26–43	427	35	24–42	< 0.001*
		Molar	413	24	15–32	410	22	13.8–30.3	< 0.001*
4 mm	AP	Anterior	87	4	2–7	87	4	2–7	0.897
		Premolar	102	3	2–6	102	3	2–6	0.875
		Molar	131	6	3–10	142	5	3–9	0.435
	RC	Anterior	155	4	2–6	155	3	1–5	0.014*
		Premolar	192	4	2–6	192	3	1.3–5	0.018*
		Molar	131	6.5	4–10	142	6	3–9	0.041*
	Total	Anterior	242	4	2–7	242	3	2–6	0.060
		Premolar	294	4	2–6	294	3	2–6	0.091
		Molar	373	6	4–10	373	6	3–9	0.035*
≥ 5 mm	AP	Anterior	21	3	1–9	15	4	1–6	0.432
		Premolar	27	4	2–9	22	2.5	2–5.5	0.104
		Molar	37	5	3–10	32	5	2.3–8	0.253
	RC	Anterior	23	3	2–6	22	2	1–3.3	0.378
		Premolar	27	3	1–5	19	2	1–3	0.452
		Molar	49	5	3–9.5	42	6.5	2.8–9	0.348
	Total	Anterior	44	3	1.3–7	37	2	1–6	0.278
		Premolar	54	3.5	1–6	41	2	1.5–4	0.085
		Molar	86	5	3–10	74	6	2.8–9	0.150
≥ 6 mm	AP	Anterior	16	2.5	2–4.5	16	2	1.3–3.8	0.326
		Premolar	26	2	1–5	33	2	1–4	0.818
		Molar	66	3	2–5	66	3	2–6	0.822
	RC	Anterior	35	2	1–4	35	2	1–3	0.401
		Premolar	26	3	1–3.5	33	2	1.5–3	0.252
		Molar	105	3	2–6	105	3	2–5	0.030*
	Total	Anterior	51	2	1–4	51	2	1–3	0.209
		Premolar	59	3	1–4	59	2	1–3	0.279
		Molar	171	3	2–6	171	3	2–5	0.066

* significant

Regardless of tooth type and BOP, there was a statistically significant decrease in the median number of sites with PPD of 0–3 mm at T2 within the both the AP group (*p* = 0.001) and RC group (*p* = 0.002). No significant change was observed in the median number of sites with PPD of 4 mm at T2 within the AP group (*p* = 0.765), whereas a significant decrease was observed in the RC group (*p* = 0.014; see Table 3).

Nevertheless, regardless of tooth type and BOP, a statistically significant reduction in sites with PPD ≥ 4 mm at T2 was found within both groups (AP: T/T2: 18 [11–31]/16 [9.3–28], *p* = 0.017; RC: T1/T2: 18 [10–28]/16 [9–26], *p* < 0.001). Additionally, a significant decrease in the median number of sites with PPD 5 mm at T2 occurred within the AP group (T1/T2: 5 [3–8]/4 [2–7], *p* = 0.030), whereas the RC group exhibited no significant change (T1/T2: 4 [2–6]/4 [2–7], *p* = 0.785). Regardless of tooth type at T1, the AP group had a significantly higher median number of sites with PPD 5 mm than the RC group (AP/RC: 5 [3–8]/4 [2–6], *p* = 0.003), but this difference was no longer present at T2 (AP/RC: 4 [2–7]/4 [2–7], *p* = 0.718). Regardless of tooth type and BOP for measurement sites with PPD ≥ 5 mm, the AP group had a higher baseline median number of affected sites compared to the controls (AP/RC: 8 [4–16]/6 [3–12], *p* = 0.002), but this difference disappeared at T2 (AP/RC: 6 [3–13]/5.5 [3–11], *p* = 0.104).

Both groups exhibited a statistically significant decrease in the number of sites with PPD ≥ 5 mm at T2 (AP: T1/T2: 8 [4–16]/6 [3–13], *p* = 0.006; RC: T1/T2: 6 [3–12]/5.5 [3–11], *p* = 0.001). For PPD ≥ 6 mm, no significant change was observed within the AP group (T1/T2: 5 [2–8]/3 [2–6], *p* = 0.119), while a significant reduction was noted in the RC group (T1/T2: 4 [2–7]/3 [2–6], *p* < 0.001). No significant intergroup differences were found in the number of sites with PPD 0–3 mm, 4 mm, ≥ 4 mm, or ≥ 6 mm at either T1 or T2 (*p* > 0.05; see Table 3).

**Table 3:** Statistical analysis of sites with pocket probing depth regardless of tooth type and regardless of bleeding on probing at T1 and T2 for air-polishing and rubber cup groups

	Group	T1			T2				
		*N*	Median	IQR	*N*	Median	IQR	*p*-value	Effect size (d)
0–3 mm	AP	152	119	97–138	152	117.5	89.3–136	0.001*	0.279
	RC	278	122	103.8–138	278	121.5	100–139	0.002*	0.184
	*p*-value		0.274			0.221			
	Effect size (*d*)		0.106			0.118			
≥ 4 mm	AP	151	18	11–31	148	16	9.3–28	0.017*	0.197
	RC	276	18	10–28	275	16	9–26	< 0.001*	0.245
	*p*-value		0.376			0.279			
	Effect size (*d*)		0.086			0.105			
≥ 5 mm	AP	137	8	4–16	127	6	3–13	0.006*	0.251
	RC	257	6	3–12	238	5.5	3–11	0.001*	0.216
	*p*-value		0.002*			0.104			
	Effect size (*d*)		0.314			0.171			
≥ 6 mm	AP	114	5	2–8	95	3	2–6	0.119	0.175
	RC	197	4	2–7	165	3	2–6	< 0.001*	0.344
	*p*-value		0.267		0.209				
	Effect size (*d*)		0.125		0.154				

* significant

### Improvement, stability, and worsening of PPD ≥ 5 mm

The proportion of sites with PPD ≥ 5 mm at T2 remained stable in 13.8% of the AP group versus 15.9% of the RC group, improved in 47.2% of the AP group versus 47.4% of the RC group, or worsened in 38.8% of the AP group versus 36.6% or the RC group, with no significant differences between groups. For clarity, ‘improved’ was defined as a reduction in PPD by ≥ 1 mm, ‘stable’ as no change in PPD, and ‘unimproved’ as an increase in PPD by ≥ 1 mm.

No significant differences between the groups were observed in the number of anterior teeth (*p* = 0.750) or premolars (*p* = 0.327) with PPD ≥ 5 mm at T2, but for molars with PPD ≥ 5 mm, a significant difference was found: The AP group had fewer stable sites (7.5% vs.17.2%) and improved sites (47.2% vs. 49.5%) but more unimproved sites (45.3% vs.33.3%) than the RC group (*p* = 0.027; see Table 4).

**Table 4:** Descriptive statistics for improved, stable and unimproved sites with different pocket probing depth measurements at T2 for air-polishing and rubber cup groups

PDD	Tooth type	Outcome at T2	Test group (AP)		Control group (RC)		Total		*p*-value	Effect size (v)
			N	%	N	%	N	%		
0–3 mm	Anterior	Stable	26	17.2	62	22.3	88	20.5	0.268	
		Improved	68	45	105	37.8	173	40.3		0.079
		Unimproved	57	37.7	111	39.9	168	39.2		
	Premolar	Stable	19	12.7	40	14.4	59	13.8	0.581	
		Improved	81	54	135	48.7	216	50.6		0.051
		Unimproved	50	33.3	102	36.8	152	35.6		
	Molar	Stable	8	5.6	20	7.5	28	6.8	0.425	
		Improved	88	61.1	145	54.7	233	57		0.065
		Unimproved	48	33.3	100	37.7	148	36.2		
≥ 4 mm	Anterior	Stable	12	12.2	23	13.3	35	12.9	0.919	
		Improved	48	49	87	50.3	135	49.8		0.025
		Unimproved	38	38.8	63	36.4	101	37.3		
	Premolar	Stable	17	14.5	25	11.7	42	12.7	0.347	
		Improved	54	46.2	116	54.5	170	51.5		0.080
		Unimproved	46	39.3	72	33.8	118	35.8		
	Molar	Stable	9	6.9	21	8.4	30	7.8	0.733	
		Improved	74	54	138	55.2	212	54.8		0.040
		Unimproved	54	39.4	91	36.4	145	37.5		
≥ 5 mm	Anterior	Stable	8	19	13	16.9	21	17.6	0.750	
		Improved	19	45.2	31	40.3	50	42		0.070
		Unimproved	15	35.7	33	42.9	48	40.3		
	Premolar	Stable	14	20.6	11	12.4	25	15.9	0.327	
		Improved	33	48.5	44	49.4	77	49		0.119
		Unimproved	21	30.9	34	38.2	55	35		
	Molar	Stable	8	7.5	32	17.2	40	13.7	0.027*	
		Improved	50	47.2	92	49.5	142	48.6		0.157
		Unimproved	48	45.3	62	33.3	110	37.7		
≥ 6 mm	Anterior	Stable	3	18.8	10	28.6	13	25.5	0.642	
		Improved	8	50	13	37.1	21	41.2		0.132
		Unimproved	5	31.2	12	34.3	17	33.3		
	Premolar	Stable	5	19.2	6	18.2	11	18.6	0.680	
		Improved	9	34.6	15	45.5	24	40.7		0.114
		Unimproved	12	46.2	12	36.4	24	40.7		
	Molar	Stable	10	15.2	16	15.2	26	15.2	0.275	
		Improved	27	40.9	55	52.4	82	48		0.123
		Unimproved	29	43.9	34	32.4	63	36.8		

AP: air-polishing group, RC: rotating rubber cup polishing group, * significant

### Furcation involvement (FI)

No significant difference was observed between the groups for multirooted teeth with FI Grade II regardless of BOP at T1 (*p* = 0.451) or T2 (*p* = 0.561). The AP group exhibited a higher number of multirooted teeth with Stage III FI regardless of BOP at T1 compared to the RC group (AP/RC: 2(1–5)/1(1–3); *p* = 0.046), but this difference was no longer present at T2 (AP/RC: 2(1–3)/1(1–3); *p* = 0.374; see Table 5).

**Table 5:** Number of multirooted teeth with a furcation involvement of stages II and III, regardless of bleeding on probing at T1 and T2 for air-polishing and rubber cup groups

	Group	T1			T2		
		*N*	Median	IQR	N	Median	IQR
FI Stage II	AP	18	2	1–3	20	1	1–2
	RC	25	2	1–3	28	1.5	1–2.8
	*p*-value		0.451			0.561	
	Effect size (*d*)		0.298			0.196	
	Total	43	2	1–3	48	1	1–5
FI Stage III	AP	13	2	1–5	16	2	1–3
	RC	18	1	1–3	24	1	1–3
	*p*-value		0.046*			0.374	
	Effect size (*d*)		0.485			0.368	
	Total	31	1	1–5	40	1.5	1–3

*significant

## Discussion

The main purpose of this longitudinal study was to compare the effect of two professional mechanical biofilm removal techniques: air-flowing using low-abrasive powders such as erythritol or glycine (AP group) and rubber cup polishing with polishing paste (RC group) in SPT over a period of at least five years at T1 and T2. Both methods demonstrated effectiveness in maintaining or improving periodontal health, though distinct differences emerged with regard to site-specific and morphological outcomes.

PMPR and the targeted subgingival debridement of residual pockets remain central elements of SPT. PMPR involves the removal of supragingival (no-)mineralized biofilm [3]. Neither the S3 guideline for treating Stages I–III of periodontitis nor the S3 guideline for treating Stage IV periodontitis specify whether manual, powered, or air-flowing instruments are preferable for PMPR and subgingival cleaning during SPT [3, 18]. A recent systematic review with meta-analysis found that repeated subgingival debridement using AP, manual, or powered instruments yielded similar clinical outcomes, with AP offering better patient comfort [19]. Another meta-analysis revealed no clinical advantage of glycine powder AP over manual or ultrasonic instruments regarding probing depth, recession, plaque index, or attachment level, though power water jet devices was perceived as less painful [20]. Similar results, demonstrating no superiority of any instrument category, also were observed in the studies by Abdulbaqi et al. [21] and Nascimento et al. [22]. The study was designed relying on the hypothesis that patients treated according to the test procedure would show a significantly lower number of periodontal pockets with PPD ≥ 5 mm after the observation period. This hypothesis had to be rejected for molars with furcation involvement and deep periodontal pockets.

Both groups experienced significant reductions in sites with PPD ≥ 4 mm and ≥ 5 mm between baseline (T1) and follow-up (T2) (*p* ≤ 0.05), suggesting that both techniques support periodontal stability when applied regularly [22]. Notably, the AP group exhibited a significant reduction in sites with PPD ≥ 5 mm (*p* = 0.006), despite presenting with higher baseline disease severity. Conversely, while the RC group also achieved a significant reduction in sites with PPD ≥ 5 mm (*p* = 0.001), this technique failed to specifically demonstrate a significant improvement at the critical 5 mm threshold (*p* = 0.785), which may reflect a plateauing of clinical gains in deeper sites. It is generally known that the number of PPDs of ≥ 5 mm is a factor for periodontal stability. Based on numerous evaluated studies, Feres et al. described a critical threshold of four sites of residual pockets that should not be exceeded to ensure long-term periodontal stability. Therefore, both techniques represent suitable options to achieve this goal, and factors such as patient preferences and clinician skills can be significantly incorporated into the decision-making process [22].

Nevertheless, the RC group exhibited a significant reduction of PPD with 4 mm from T1 to T2 (*p* = 0.014) in contrast to the AP group (*p* = 0.765). This could indicate that the treatment in the RC group was possibly more efficient or that there are differences in the healing response between the two groups. It is known that subgingivally applied powders can directly influence gingival fibroblasts, whereby a cytotoxic effect of erythritol powder due to the CHX component has already been demonstrated [23]. It should be noted that the vigorous nature of PMPR may result in irritation or inflammation of the gums, which could be identified as a reason for wound healing disorders. However, according to current evidence, patient discomfort and pain indicated that treatment with AP might be less painful than, for example, ultrasonic scaling [20]. On the one hand, at PPD of 5 mm, the AP group exhibited a significant reduction (*p* = 0.030), while no changes occurred in the RC group. This could indicate that deeper periodontal pockets in the AP group were more responsive to treatment, which is in line with the general knowledge of the impact of periodontal treatment in deeper pockets compared to lower pocket depths [24]. On the other hand, PPD values were significantly higher in the AP group than in the RC group at T1 but no longer at T2, which leads to the conclusion of a delayed but ultimately comparable healing reaction. Moreover, this observation could be due to the respective system’s advantages and disadvantages.

Subgroup analysis revealed that molars with PPD ≥ 5 mm responded more favorably to RC treatment than to AP. The AP group demonstrated a significantly higher proportion of deteriorated molar sites with PPD ≥ 5 mm (45.3%) than the RC group (33.3%; *p* = 0.027). This could be due to the fact that the powder-water jet, as routinely used in the current investigation without special subgingival instrument approaches, does not penetrate further than approximately 5 mm, coupled with the much more complicated morphology of furcations and multirooted molars. Petersilka et al. [25] supports this outcome, reporting that the cleaning effectiveness using AP decreased to approximately 30% of the cleaning performance of RC at PPD ≥ 5 mm. Again, a further study revealed that both methods of PMPR are effective in removing biofilm from the furcation area, but conventional mechanical debridement with powered scalers was even more effective [26]. Moreover, the RC group achieved a significant reduction in molar sites with PPD ≥ 6 mm (*p* < 0.001), while the AP group did not (*p* = 0.119). This highlights a possible clinical advantage of RC in managing advanced periodontal pockets in posterior dentition, a finding consistent with previous evidence suggesting superior debridement of furcation sites via conventional mechanical methods [12, 26].

Our data further demonstrate no significant difference between the number of multirooted teeth with an FI Stage II at T1 (*p* = 0.451) or T2 (*p* = 0.561) in the two groups, except at T1 (*p* = 0.046), where the AP group exhibited a significantly higher median number of multirooted teeth with an FI Stage III regardless of BOP than the RC group, but no longer at T2 (*p* = 0.374). In SPT, AP treatment generally leads to a significant reduction in FI Stage III over a period of at least five years, from T1 to T2 in this study (*p* = 0.046) [26, 27]. Nevertheless, in the AP group, molars with FI Stage III and PPD of ≥ 5 mm revealed a deterioration in PPD (*p* = 0.027) compared to the RC group molars [12, 26]. Petersilka et al. were even able to demonstrate that, in SPT, it could be contraindicated to treat furcation defects with AP only, and that AP does not improve the clinical outcome [12]. This apparent contradiction—AP showing overall comparable outcomes, yet RC performing better in molars with furcation-involvement—may be explained by anatomical and technical limitations [26]. Ultrasonic scaler combined with AP devices may not effectively penetrate complex furcation areas beyond 5 mm, whereas mechanical plaque removal via combined sonic scalers/curettes and rotating rubber cup/brushes offers superior access in these challenging sites. Therefore, while AP may suffice for most sites, RC appears more reliable for posterior teeth with advanced furcation defects, at least according to the current state of technical development.

Similar to Nascimento et al. [28], no significant difference in treatment time between the AP and RC groups (*p* = 0.378) was evident. However, the study demonstrated that AP takes approximately 2 min longer to administer than RC, a finding that may be clinically negligible. It should also be noted that the recorded time encompassed the entire SPT session and was not limited to the specific use of AP or RC. Conversely, a number of studies have indicated that AP is less time-consuming than RC [29–32]. Therefore, it is assumed that when the technique is embedded within a structured treatment process such as SPT, the time saved by using AP is often reallocated to other valuable activities, such as patient motivation, instruction in home oral hygiene, and education about additional risk factors. This redistribution improves patient adherence to SPT protocols, which is a crucial factor for the long-term maintenance of periodontal health. Thus, the time difference should not be regarded as a disadvantage of AP; rather, the overall benefit lies in improved plaque removal combined with elevated patient engagement and adherence to therapy [25–29]. Furthermore, there are currently no national or international guidelines for the subgingival use of powder-water jet devices in SPT for all stages of periodontal therapy [3], reflecting a need for further high-quality randomized trials to define appropriate indications and standardized protocols.

### Clinical implications

Given the inferior performance of AP in molars with advanced furcation involvement, clinicians may consider favoring conventional RC polishing in such cases during SPT. However, for non-molar teeth or sites without furcation-involvement, AP remains a time-efficient and patient-friendly alternative. This underlines the importance of tailoring biofilm management to site-specific anatomical conditions and individual risk profiles.

### Limitations

Confounding variables may have influenced and limited the results of this study. The examinations (e.g., measurements of PPD or FI) and treatment were performed by different expert staff members. Although all dental examiners have several years of experience, an individual’s skill in handling various instruments can influence their efficiency. Thus, patients’ retention of mineralized and non-mineralized deposits and biofilm could have varied. Despite the fact that all expert staff members had an airflow device at their disposal during SPT, it can be assumed that not all used it according to the same selection principles (e.g., due to individual preferences or specific patient wishes). However, an advantage of this was an automatic division of the treated patients into test versus control group without creating a selection bias through dental examiners. Due to the real-world observational nature of this study, randomization and full blinding of examiners were not feasible. However, procedural independence was maintained to a reasonable extent; while dental hygienists performed the supportive periodontal therapy, periodontal examinations were carried out separately by dentists. Although awareness of the treatment modality among examiners cannot be excluded entirely, this structure likely helped minimize the risk of detection bias. In addition, group allocation was not disclosed to the statistician during data analysis, further reducing the risk of analytic bias. To implement a quality standard within the university setting, the expert staff members have the opportunity to take part in certified training courses. Until 2018, all employees in the functional area of periodontology had received instruction and training on the devices from EMS (Switzerland) and NSK (NSK Europe GmbH, Eschborn, Germany). In contrast to studies employing a single, standardized device, the use of different systems may have influenced outcomes, especially considering that handpiece-mounted units are known to show performance variability during prolonged use [33].

The average number of treatments per patient over the five-year observation period was nine, indicating that most participants received consistent long-term maintenance care. Neverthelss, individual risk factors — such as poor oral hygiene or smoking — may have substantially influenced a considerable impact on plaque levels and PPDs, particularly under low-frequency maintenance intervals [34]. As no detailed data on changing of smoking status (e.g. level of reduction, temporarly smoking stopps) or plaque levels as a result of a change in personal (oral) behaviors were documented, the generalizability and clinical applicability of the results are limited and should be considered when interpreting the findings. Both groups demonstrated a deterioration in plaque index, which is likely attributes to the particulary pronounced impact of intensive oral hygiene instruction during APT and the APT itself at the onset of SPT. Nonetheless, the uniform recruitment strategy likely ensured a balanced distribution of these factors across the study groups, thereby reducing the potential for confounding.

## Conclusion

Both methods of PMPR, AP and RC, were effective in maintaining or improving periodontal health during supportive periodontal therapy. While overall outcomes were comparable, conventional polishing with rotating rubber cups or brushes demonstrated superior results in molars and deep pockets, especially in sites with furcation involvement. These results underline the need for a diagnosis- and patient-individualized approach in SPT.

## Data Availability

The datasets used and analyzed during the current study are not publicly available due to [national data protection law] but are available from C. Graetz on reasonable request.

## Abbreviations

AP: Air-polishing (test group)
APT: Active periodontal treatment
BOP: Bleeding on probing
FI: Furcation involvement
PPD: Pocket probing depths
PMPR: Professional mechanical plaque reduction
RDA: Relative dentin abrasion
RC: Rubber cup (control group)
SPT: Supportive periodontal therapy
